# A review on anxiety and sleep

**DOI:** 10.6026/973206300210965

**Published:** 2025-05-31

**Authors:** Shivangi Saxena

**Affiliations:** 1 Department of Internal Medicine, Raja Rajeswari Medical College & Hospital, Bangalore, India

**Keywords:** Anxiety, sleep disturbances, insomnia

## Abstract

The relationship between sleep and anxiety throughout childhood, adolescence and adulthood is of interest to highlight the prevalence of anxiety as a disorder. Both children and parents report higher sleep disturbances. However, known data show similar total sleep time between anxious and non-anxious children. There is a positive correlation between nightmares and trait anxiety among school-aged children with no significant differences after a week of evaluation. In adolescents, anxiety is associated with longer sleep onset times and increased REM (Rapid Eye Movement) sleep. Nonetheless, the connection between sleep and anxiety in adults has been less explored despite high occurrence rates. Moreover, poor sleep quality and disturbances are prevalent in the elderly. Furthermore, a link between trait anxiety and increased wake after sleep onset, particularly in individuals with primary insomnia is known.

## Background:

A variety of symptoms and disorders are included in sleep disturbances, such as hypersomnia, insomnia, circadian rhythm disturbance, extrinsic sleep disorders and excessive daytime sleepiness. It is known that there is a presence of high comorbidity rates between sleep disturbances and anxiety, but their contributions vary among different anxiety disorders. Some studies have found a distinct relationship wherein anxiety foresees sleep disturbance but not the other way around [1]. Dissatisfaction with either the quality or quantity of sleep is associated with difficulty for initiating and maintaining sleep as well as early-morning awakening. This is commonly defined as insomnia [2]. Sleep disturbances are indications for emotional and behavioural issues, as well as linked to psychiatric disorders throughout life, playing a role as both a symptom and a precursor [3]. About 4% of people worldwide are thought to suffer from anxiety disorder and it is the most prevalent mental disease globally, affecting 301 million individuals in 2019 [4]. The interrelation between anxiety and sleep disturbances is perceived to be bidirectional according to some studies [5]. Therefore, it is of interest to discuss the relationship between sleep and anxiety.

## Materials and Methods:

In conducting a narrative review on the relationship between anxiety and sleep, a systematic search was conducted on PubMed utilizing the keywords "anxiety AND sleep NOT alcohol NOT cannabis*".

## Eligibility criteria:

## Inclusion criteria:

The inclusion criteria focused on studies published from 2013 to 2024 and free full-text availability. The review incorporated systematic reviews (with or without meta-analysis), clinical trials, related narrative reviews, and select surveys. These parameters resulted in 10,081 articles, of which 1034 research articles were assessed.

## Exclusion criteria:

Out of the 1034 research articles, four exclusion criteria were implemented to refine the search to include relevant articles. This included the exclusion of any articles that did not focus on the relationship between sleep and anxiety in humans, assessed sleep and anxiety in a particular group (*e.g.* doctors, nurses, students), assessed sleep and anxiety in a particular disease (*e.g.* COVID, fibromyalgia) or assessed a treatment and its effect on sleep and anxiety (*e.g.* drugs, yoga). After the exclusion criteria, 43 articles were identified, of which 17 papers were included in the current narrative review.

## Discussion:

## Sleep and anxiety in childhood:

In childhood, a healthy sleep pattern includes regular bedtimes and wake-up times, which results in sufficient sleep and parent-child interactions, which are positive in nature. However, children suffering from Generalized Anxiety Disorder (GAD) persistently report higher rates of sleep disturbances and substantially higher total sleep problems are reported by parents of children afflicted with GAD when compared to parents of control. A study conducted by Alfano *et al.* used one week's worth of actigraphy data to scrutinise its relation with subjective reports given by school-aged children with GAD and the healthy control group. It showed that parents of children estimated comparable subjective sleep patterns such as bedtimes, total duration of sleep and wake-up time in both groups in objective comparisons of sleep [6]. Children who are anxious have been noted to showcase anxiety related to sleep, resistance to going to bed, and higher levels of somatic and cognitive pre-sleep arousal than children who are not anxious. A positive correlation is seen in school-aged children between trait anxiety and nightmares, including higher trait anxiety scores in kids who rate their nightmares as more frightening [7]. In another study that monitored anxious and non-anxious children in their homes with the help of poly-somno-graphy (PSG), it was noted that there was no variation between the two groups in their sleep, with the exception of significantly improved sleep efficiency and lesser Rapid Eye Movement (REM) in children suffering from GAD [8]. In another study by Cohodes *et al.* a relative increase in sleep function was measured by actigraphy in children with higher anxiety-related symptomatology compared to controls. This showcases that increased sleep as a clinical marker could be used potentially to detect the onset of anxiety [9]. However, a prospective evaluation of nightmares failed to showcase a difference between anxious and non-anxious groups when monitored for a week and this conclusion is backed by similar actigraphy total sleep time and awakenings at night [7]. The distinct types of issues displayed by children with GAD, such as requests to co-sleep, attempts to delay bedtime, expression of nighttime fears, and complaints of nightmares may explain the disparity between children's sleep patterns and endorsement of sleep problems. However, despite these behaviors occurring in connection to sleep, they may not change the assessed sleep parameters [6].

## Sleep and anxiety in adolescents and teenagers:

Sleep plays a meaningful role in the initiation, progression and continuation of mood disorders in this time period. According to research as there are already existing susceptibilities in systems of emotion and sleep exist in youth with anxiety, placing them at an increased risk. Additionally, in the community children and adolescents have a high prevalence of delayed sleep phase, insomnia and hypersomnia [10]. In youth with GAD, a longer onset of sleep latency, as well as an increased entry into REM sleep, was found with the help of PSG in a sleep lab when compared to non-anxious children. However actigraphy continued to show an absence of sleep differences between the two groups [3]. In a study by Mesa *et al.* a multimodal method was used to determine the quality of sleep in adolescents with Social anxiety disorder over the course of seven days, which showed that they attained 7.36 hours of sleep on average every night, and 94.29% did not complain of sleep difficulties which was in line with the control group [11].

## Sleep and anxiety in adults:

The relationship between sleep and anxiety has been neglected in this age group, especially considering the fact that adults in mid to late life have high rates of both sleep disturbance and anxiety [12]. In a systematic review conducted by Alvaro *et al.* most articles reported at least one compelling relationship between sleep disturbance and anxiety. However, more studies are needed to draw a definitive relationship between the two as current evidence showcases a bidirectional relationship between anxiety and insomnia, according to four longitudinal studies [1]. Additionally, a negative association is seen in late life anxiety and sleep due to greater sleep fragmentation, poorer sleep efficiency and poorer self-reported sleep duration when compared to adults with less anxiety [12]. Accounting for sex differences, higher levels of anxiety are seen in women triggered by sleep loss compared to men and a decrease in the volume of grey matter is seen in the anterior insula and lateral orbitofrontal cortex in women, leading to anxiety. However, it predicts resilience in men with great discrimination accuracy [13]. On the contrary, in both men and women, the anxiogenic impact of sleep loss was predicted by the grey matter volume in the ventromedial prefrontal cortex [13]. In a magnetic resonance imaging study by Wang *et al.* it was noted that after a whole-brain regression analysis, a thin cortex in the left superior temporal sulcus (STS) is associated with worse sleep quality and the relation between the emotional symptoms and the quality of sleep was mediated by the thickness of the left STS [14]. In another MRI study by Li *et al.* it was noted that altered hypothalamic resting state functional connectivity are bi-directionally related to poor sleep quality in anxiety among men [15].

## Sleep and anxiety in the elderly:

In a study conducted by Gould *et al.* it was demonstrated that affective and somatic anxiety clinical features were the most robustly associated with global sleep quality in the elderly which was calculated with the help of the Pittsburgh Sleep Quality Index (PSI). PSI measures the quality of sleep for the past month by seven components such as daytime dysfunction, sleep duration, sleep quality, sleep efficiency, sleep latency, sleep disturbances and the use of sleep-related medicines which is added to derive the global score. Additionally, daytime sleepiness was noted to be related to increased somatic and cognitive anxiety clinical features. However, this study lacked an objective measure through actigraphy and PSG of said sleep disturbances [17]. Furthermore, dysfunctional attitudes and beliefs about sleep, rooted in incorrect cognitive concepts about insomnia's causes and consequences, as well as unrealistic expectations of sleep, can produce the undesirable effect of delaying sleep. Efforts to suppress these intrusive thoughts can produce the undesirable effect of delaying sleep. Individuals affected with psychiatric disorders have more flawed beliefs about sleep when compared to healthy individuals. Anxiety is linked to dysfunctional beliefs about sleep and this translates to the elderly who sleep poorly have been shown to have flawed perceptions about sleep compared to those who sleep well [16]. The odds of being afflicted with anxiety are higher if an individual believes in statements such as "When I find it hard to fall asleep, I should stay in bed and keep trying to sleep" and has apprehension about the idea of sleeping at bedtime and there is a connection between the probability of developing an anxiety disorder and not agreeing with the statement, "It is normal not to sleep as well as we get older [15]. It was demonstrated in a study that trait anxiety was linked to higher actigraphy measured Wake after Sleep Onset in a sample of elderly with primary insomnia [18].

## Conclusion:

It is impossible to accurately depict a genuine relationship between sleep disturbances and anxiety due to the small sample size studied hence, longer longitudinal studies are required to determine reciprocal relationship between them. Further, many relevant sleep issues like prolonged sleep, overtiredness, resistance to going to bed, sleep-anxiety, parasomnias, sleep-disordered breathing *etc.* are not consistently assessed. Therefore, future studies should assess sleep in anxious individuals with the help of subjective and objective parameters such as actigraphy and PSG to better differentiate between mal-adaptive thoughts regarding sleep in anxious individuals versus maladaptive sleep patterns in the same group.
